# Difference in Cerebral Circulation Time between Subtypes of Moyamoya Disease and Moyamoya Syndrome

**DOI:** 10.1038/s41598-017-02588-1

**Published:** 2017-05-31

**Authors:** Kaijiang Kang, Jingjing Lu, Dong Zhang, Youxiang Li, Dandan Wang, Peng Liu, Bohong Li, Yi Ju, Xingquan Zhao

**Affiliations:** 1 Department of Neurology, Beijing Tiantan Hospital, Capital Medical University, China National Clinical Research Center for Neurological Diseases, Center of Stroke, Beijing Institute for Brain Disorders, Beijing, China; 2 Department of Neurosurgery, Beijing Tiantan Hospital, Capital Medical University, Beijing, China; 3 Department of Neurosurgery, Beijing Neurosurgical Institute, Capital Medical University, Beijing, China

## Abstract

In this study, we evaluated the differences in hemodynamics between hemorrhagic and non-hemorrhagic moyamoya disease (MMD) and moyamoya syndrome (MMS) by measuring cerebral circulation time (CCT). This case-control study included 136 patients with MMD or MMS diagnosed between April 2015 and July 2016 at Beijing Tian Tan Hospital. Each hemisphere was analyzed separately. The difference in clinical, radiological characteristics and CCT between subtypes of MMD and MMS were analyzed statistically. The results showed that total CCT between hemorrhagic and non-hemorrhagic sides was not statistically different (16.55 s vs. 16.06 s, P = 0.562). The cerebral filling circulation time (CFCT) of hemorrhagic sides was significantly shorter than that of non-hemorrhagic sides (4.52 s vs. 5.41 s, P < 0.001), and the cerebral venous circulation time (CVCT) of hemorrhagic sides was significantly longer than that of non-hemorrhagic sides (12.02 s, vs. 10.64 s, P < 0.001). The ratio of CFCT to CVCT (F-V ratio) was inversely correlated with the possibility of hemorrhagic stroke. Therefore, we conclude that the rapid filling and poor venous drainage of cerebral circulation are likely risk factors of hemorrhagic stroke secondary to MMD or MMS. The F-V ratio can be used to identify individuals at high risk of hemorrhagic stroke.

## Introduction

Moyamoya disease (MMD) is an uncommon cerebrovascular disease with unknown etiology; it is called moyamoya syndrome (MMS) if associated with underlying disease^1–4^. The prevalence of MMD is high in East Asian countries such as Japan, Korea, and China^2, 5–14^. As the disease progresses, MMD is divided into six stages (Suzuki stage) according to cerebral angiographic findings^2^. During the disease course, there may be varying degrees of cerebral ischemia (often has a relatively benign prognosis) or intracranial hemorrhage (one of the main factors leading to acute death and disability). In the Hokkaido area of Japan, 21% of patients with MMD experienced intracranial hemorrhage from 2002 to 2006^8^, and in Korea, the proportion of hemorrhagic MMD increased up to 42.4%^14^. Thus it is important to identify the hemodynamic differences between hemorrhagic and non-hemorrhagic MMD or MMS. It is generally believed that ischemic stroke secondary to MMD or MMS is due to gradual occlusion of the internal carotid artery (ICA), but external carotid artery (ECA) and posterior cerebral artery (PCA) have not yet fully compensated for the impaired blood supply, while intracranial hemorrhage mainly occurs due to the rupture of abnormal moyamoya vessels and dilated collateral vessels^5, 15–19^. However, the mechanisms underlying ischemic or hemorrhagic stroke secondary to MMD are confined to the structures, such as dilation at the junction of the ICA–posterior communicating artery and artery aneurysms^15–19^. Cerebral vessels work together to service the cells of the brain, and any cerebrovascular abnormality can result in cerebral circulatory abnormalities. These structural features do not reflect the comprehensive cerebral blood flow, and changes in structure are largely due to hemodynamic abnormalities. Thus, it is imperative to determine the hemodynamic changes that occur in MMD or MMS.

In this study, we evaluated the differences in hemodynamics between patients with hemorrhagic and non-hemorrhagic MMD or MMS by measuring cerebral circulation time (CCT).

## Materials and Methods

### Patient selection

This case-control study included 136 patients, aged 5–65 years, who were diagnosed with MMD or MMS (according to the diagnostic guidelines proposed by the Ministry of Health and Welfare of Japan ref. 20) between April 2015 and July 2016 at Beijing Tian Tan Hospital. The study was performed according to the Declaration of Helsinki guidelines, and written informed consent was obtained from all participants. The patients in our study only underwent standard treatment without additional interventions for research purposes, so no formal ethics approval was required. All of the patients underwent digital subtraction angiography, at least 3 months after intracranial hemorrhage for hemorrhagic patients, at Beijing Tian Tan Hospital to confirm the diagnosis. Patients in the acute or subacute phase of stroke, which may influence cerebral hemodynamics, were excluded. Each hemisphere was analyzed separately, and postoperative hemispheres of revascularization were not included in this study. We used PASS 11 software to calculate the required sample size (42 hemorrhagic hemispheres and 126 non-hemorrhagic hemispheres) according to the two-sample t-test power analysis, based on our preliminary pilot study of 85 patients (data not published).

### Neuroimaging

Intracranial hemorrhage was diagnosed with computed tomography (CT), and cerebral infarction was diagnosed with magnetic resonance imaging. Each hemisphere (hemorrhagic or non-hemorrhagic hemisphere) was analyzed separately (71 hemorrhagic hemispheres and 178 non-hemorrhagic hemispheres) including 136 ischemic hemispheres and 42 asymptomatic hemispheres. During digital subtraction angiography (DSA) procedures, a 5 F angiocatheter was placed at the C1 segment of the ICA (corresponding to the second cervical vertebra) and the V1 segment of the vertebral artery. The imaging parameters were 4 frames/s with injection (using a power injector, pressure was 300 psi/kg) of 5 mL (3 mL/s) contrast medium for all series in all of the subjects in a single-plane angiographic machine (Artis zee floor, Siemens AG, Germany). During the angiography procedure, CCT on the lateral view of the ICA was calculated. Neuroimaging was performed by one neurologist and two trained neuroradiologists, who were blinded to the types of MMD or MMS, and who were responsible for assessing all of the neuroimaging variables used in the study. The average of the data was used to reduce the impact of subjective factors.

### Evaluation of Suzuki stage and vascular compensation

According to the manifestation of DSA, all of the included hemispheres were categorized into six stages, based on Suzuki stage^2, 21^. The compensation of the anterior choroidal artery (AChA), posterior communicating artery (PComA), posterior cerebral artery (PCA), and external carotid artery (ECA), was evaluated using a previously reported methodology^15, 22^.

### Evaluation of cerebral blood filling and cerebral venous drainage

**Figure 1:**
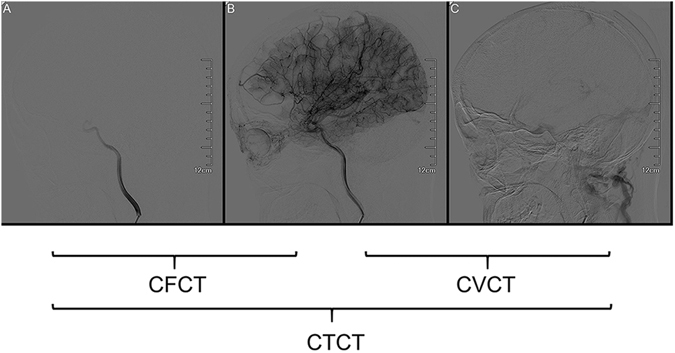
The schematic diagram of measurement of cerebral circulation time. The lateral view of internal carotid artery. CTCT: The time from appearance of the C4 segment of ICA to disappearance of the sigmoid sinus; CFCT: The time from appearance of the C4 segment of the ICA to maximum intensity of contrast; CVCT: The time from maximum intensity of contrast to disappearance of the sigmoid sinus.

We measured the CCT of 110 patients (181 hemispheres), including 43 hemorrhagic hemispheres and 138 non-hemorrhagic hemispheres. We evaluated the cerebral blood filling and cerebral venous drainage by measurement of CCT in the lateral view of the ICA. The time from appearance of the C4 segment of the ICA to disappearance of the sigmoid sinus was defined as total CTT, which was divided into cerebral filling circulation time (CFCT, from appearance of C4 segment of the ICA to maximum intensity of contrast), and cerebral venous circulation time (CVCT, from maximum intensity of contrast to disappearance of the sigmoid sinus) (Fig. 1). We used CFCT to represent cerebral blood filling or perfusion, and CVCT represented cerebral venous drainage.

### Statistical analysis

The statistical analysis was performed using a commercially available statistical software package (SPSS for Windows, version 19.0, IBM-SPSS, Chicago, IL, USA). Quantitative variables were analyzed using t-test (normal distribution) or Mann–Whitney U test (skewed distribution). Chi-squared test and logistic regression were used to analyze the differences of clinical and radiological characteristics (categorical variables) between hemorrhagic and non-hemorrhagic MMD. Receiver operating characteristic (ROC) curve analysis was used to predict hemorrhagic stroke in patients with MMD or MMS. P values less than 0.05 were considered statistically significant.

## Results

### General characteristics

The study included 136 patients (249 hemispheres) with MMD or MMS (19 cases with unilateral pathology and 4 hemispheres with revascularization), containing 71 hemorrhagic hemispheres and 178 non-hemorrhagic hemispheres (including 136 ischemic hemispheres and 42 asymptomatic hemispheres). The ratio of female to male patients was not significantly different between the hemorrhagic group and non-hemorrhagic group (1.4:1 vs. 1:1; P = 0.191). The ratio of adults (≥18 years old) to children (<18 years old) patients was also not significantly different between the hemorrhagic group and non-hemorrhagic group (23:1 vs. 6:1; P = 0.128) (Supplementary Table S1). For intracranial hemorrhage, the most frequent hemorrhagic disorder was intraventricular hemorrhage (n = 35, 49.3%), followed by cerebral hemorrhage with intraventricular hemorrhage (n = 14, 19.7%) and intracerebral hemorrhage (n = 14, 19.7%); subarachnoid hemorrhage was relatively rare (n = 8, 11.3%). There are 158 hemispheres with varying degrees and sites of cerebral infarction, 139 (88.0%) involving the frontal cortex or subcortical white matter (white matter involvement was most common), 22 (13.9%) involving the parietal lobe, 20 (12.7%) involving the temporal lobe, 16 (10.1%) involving the occipital lobe, and 30 (19.0%) involving the basal ganglia region (Supplementary Table S2). Cerebral angiography revealed that the PCA was involved in 38 cases (27.9%), characterized by varying degrees of stenosis or occlusion of the PCA, or formation of abnormal moyamoya vessels.

### Suzuki stage, vascular compensation and intracranial aneurysm

**Figure 2:**
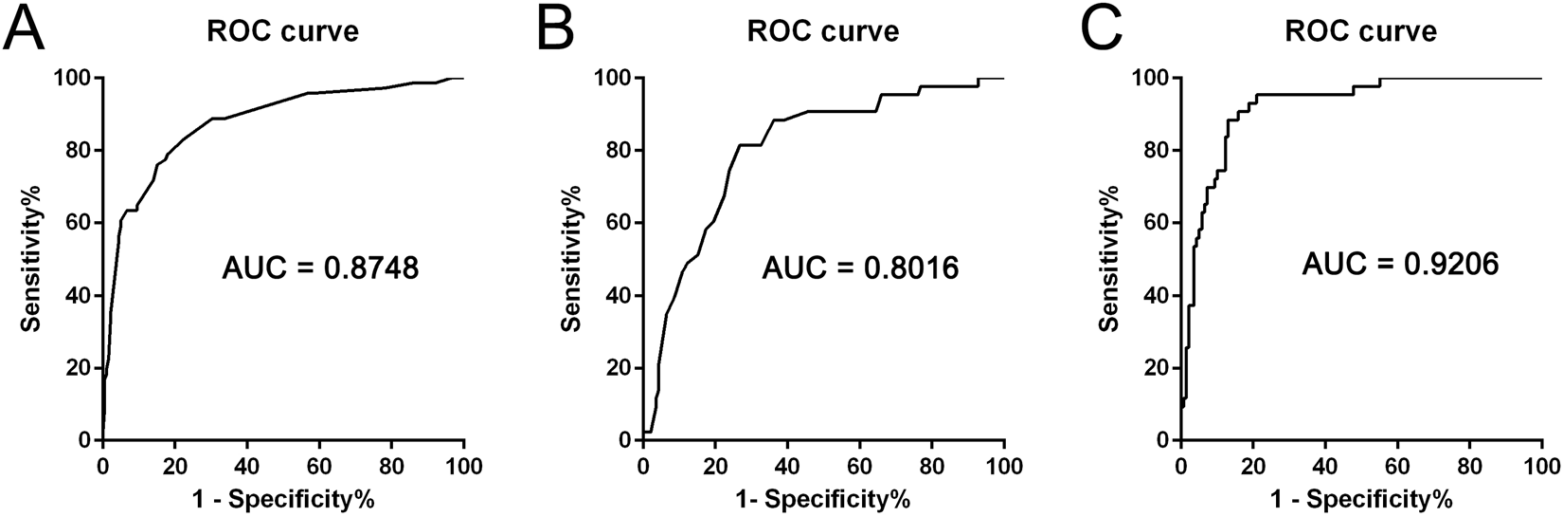
The ROC curve analysis of different indexes for differentiating hemorrhagic MMD or MMS from non-hemorrhagic MMD or MMS. (**A**) ROC curve analysis of age, gender, Suzuki stage (Suzuki 3–4), dilation of AChA (Grade 2) and PComA (Grade 2), and aneurysm for predicting hemorrhagic MMD or MMS (P < 0.0001, AUC = 0.8748). (**B**) ROC curve analysis of F-V ratio (the ratio of CFCT to CVCT) for predicting hemorrhagic MMD or MMS (P < 0.0001, AUC = 0.8016, best cut-off point 0.4344; sensitivity 81.4%, specificity 73.2%). (**C**) ROC curve analysis of the F-V ratio, combined with age, gender, Suzuki stage (Suzuki 3–4), dilation of AChA and PComA and aneurysm, for predicting hemorrhagic MMD or MMS (P < 0.0001, AUC = 0.9206).

The proportion of hemorrhagic hemispheres in Suzuki stages 3 and 4 (moyamoya vessels are most substantial) was significantly higher than that in other stages (47.7%, 34.8%, respectively). The distribution of Suzuki stages, grades of AChA, PComA, PCA, and ECA, and associated aneurysms in each hemispheric group is listed in Supplementary Table S1. ROC curve analysis of age, gender, Suzuki stage (Suzuki 3–4), dilation of AChA (Grade 2), and PComA (Grade 2), and aneurysms for predicting hemorrhagic MMD or MMS showed that the area under curve (AUC) was 0.8748 (P < 0.0001) (Fig. 2).

### CCT

We measured CCT (in the lateral view of the ICA of the DSA) of 110 patients (181 hemispheres), including 43 hemorrhagic hemispheres and 138 non-hemorrhagic hemispheres. The results showed that total CCT was not statistically different between the hemorrhagic and non-hemorrhagic group (16.55 s vs. 16.06 s, P = 0.562). The CFCT of the hemorrhagic group was relatively shorter than that of the non-hemorrhagic group, with a statistical significance (4.52 s vs. 5.41 s, P < 0.001, odds ration [OR] = 0.201), while the CVCT of the hemorrhagic group was relatively longer than that of the non-hemorrhagic group, also with statistical significance (12.02 s vs. 10.64 s, P < 0.001, OR = 1.765) (Table 1). The differences in CCT between hemorrhagic and non-hemorrhagic MMD in different demographic groups also exhibited this phenomenon, apart from the children, among which there were only three hemorrhagic hemispheres (Table 1). Subgroup analyses of different Suzuki stages showed that this phenomenon was primarily apparent in the hemispheres between Suzuki 3 and Suzuki 4, with the exception of the early stages (Suzuki 1 to 2) or late stages (Suzuki 5) (Table 1).

**Table 1:** CCT of Hemorrhagic and non-Hemorrhagic Hemisphere in Different Demographic Groups.

	CCT (95% CI)		P value	OR
	Hemorrhagic (s)	non-Hemorrhagic (s)		
Total patients				
CFCT (ICA)	4.52 (4.22–4.82)	5.41 (5.14–5.68)	<0.001	0.201
CVCT (ICA)	12.02 (11.17–12.88)	10.64 (10.28–11.01)	<0.001	1.765
CTCT (ICA)	16.55 (15.50–17.59)	16.06 (15.52–16.60)	0.562	
Male				
CFCT (ICA)	4.42 (4.06–4.78)	5.69 (5.31–6.07)	0.005	0.120
CVCT (ICA)	12.29 (11.10–13.47)	10.67 (10.22–11.13)	0.016	1.813
CTCT (ICA)	16.70 (15.45–17.95)	16.36 (15.68–17.04)	0.280	
Female				
CFCT (ICA)	4.59 (4.13–5.06)	5.12 (4.75-5.50)	0.001	0.260
CVCT (ICA)	11.84 (10.57–13.10)	10.61 (10.04–11.19)	0.008	1.547
CTCT (ICA)	16.43 (14.79–18.07)	15.75 (14.91–16.59)	0.740	
Adults (≥18 y)				
CFCT (ICA)	4.57 (4.26–4.88)	5.46 (5.17–5.75)	<0.001	0.238
CVCT (ICA)	11.89 (11.00–12.77)	10.71 (10.31–11.11)	<0.001	1.583
CTCT (ICA)	16.45 (15.35–17.55)	16.17 (15.58–16.76)	0.525	
Children (<18 y)				
CFCT (ICA)	3.90 (1.95–5.85)	5.18 (4.46–5.91)	0.052	
CVCT (ICA)	13.87 (7.35–20.39)	10.34 (9.47–11.21)	0.251	
CTCT (ICA)	17.77 (9.86–25.68)	15.52 (14.19–16.85)	0.052	
Suzuki 1–2				
CFCT (ICA)	4.10 (3.55–4.65)	4.05 (3.77–4.33)	0.914	
CVCT (ICA)	11.42 (10.01–12.83)	10.13 (9.44–10.81)	0.362	
CTCT (ICA)	15.52 (14.18–16.86)	14.18 (13.33–15.02)	0.475	
Suzuki 3–4				
CFCT (ICA)	4.57 (4.19–4.96)	5.80 (5.46–6.14)	<0.001	0.097
CVCT (ICA)	12.70 (11.65–13.75)	10.92 (10.40–11.45)	<0.001	2.404
CTCT (ICA)	17.27 (15.92–18.63)	16.74 (15.97–17.50)	0.226	
Suzuki 5				
CFCT (ICA)	4.69 (3.81–5.57)	6.55 (5.92–7.19)	0.041	0.336
CVCT (ICA)	10.46 (7.90–13.02)	10.64 (9.91–11.38)	0.156	
CTCT (ICA)	15.15 (12.06–18.25)	17.20 (16.23–18.16)	0.156	

Logistic regression (multivariate analysis) was used to analyze the differences of cerebral circulation time between hemorrhagic and non-hemorrhagic MMD in different demographic groups.

**Figure 3:**
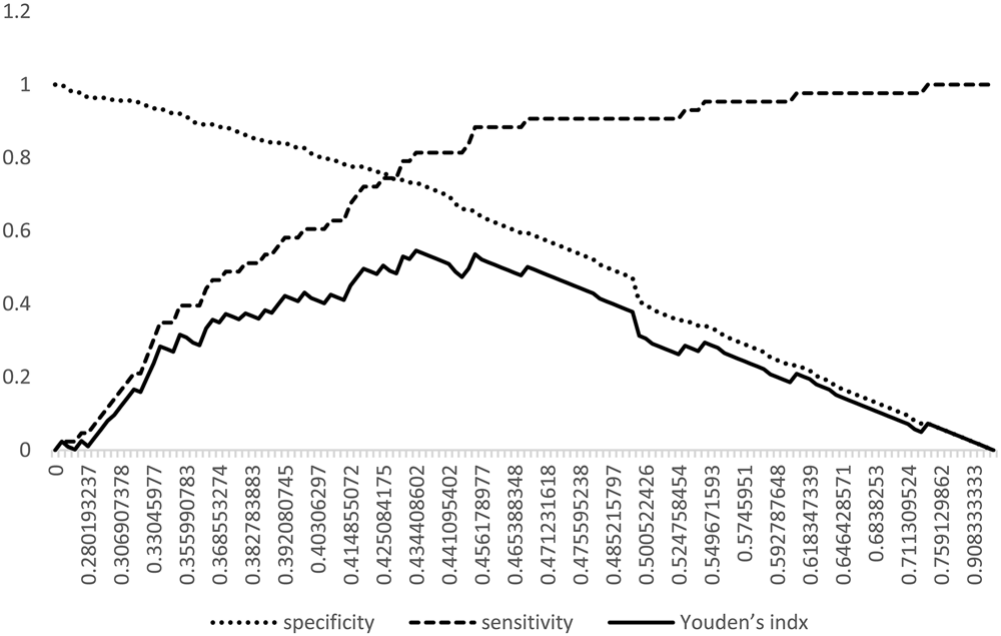
ROC curve analysis of the F-V ratio for predicting hemorrhagic MMD or MMS. ROC curve analysis of F-V ratio for predicting hemorrhagic MMD or MMS shows that the best cut-off point for predicting hemorrhagic MMD or MMS is 0.4344 (sensitivity = 81.4%, specificity = 73.2%, AUC = 0.8016), according to Youden’s index.

To diminish the interaction between CFCT and CVCT, or other influential factors of CCT (e.g., technical factors, heart rate, and blood pressure), the ratio of CFCT to CVCT (F-V ratio) was used to analyze the influence of CCT on subtypes of stroke associated with MMD or MMS. The results showed that the F-V ratio of hemorrhagic hemispheres was comparatively lower than non-Hemorrhagic hemispheres in different demographic groups (Table 2). Analysis of the F-V ratio between hemorrhagic sides and non-hemorrhagic sides in the same patient (19 patients) also showed this phenomenon (Supplementary Table S3). The results also showed that the F-V ratio, adjusted by age, gender, Suzuki stage, dilation of AchA, Pcom, and aneurysm, was of great significance for predicting hemorrhagic MMD or MMS (Table 3). In addition, the F-V ratio was inversely correlated with the possibility of hemorrhagic stroke, and the different cut-off points of the F-V ratio and corresponding sensitivity, specificity, and positive and negative predictive values for predicting hemorrhagic stroke in patients with MMD or MMS is listed in Table 4. The ROC curve analysis of the F-V ratio showed that the best cut-off point for differentiating hemorrhagic MMD or MMS from non-hemorrhagic type was 0.4344 (P < 0.0001, AUC = 0.8016, sensitivity = 81.4%, specificity = 73.2%), according to Youden’s index (Figs 2 and 3). Using logistic regression and ROC curve analysis, a comparison was done between a model only using structural metrics and a model with structural metrics and the F-V ratio for predicting hemorrhagic MMD or MMS. The results are shown in Table 5 and Fig. 2.

**Table 2:** The F-V Ratio of Hemorrhagic and non-Hemorrhagic Hemisphere in Different Demographic Groups.

	F-V Ratio (95% CI)		P value
	Hemorrhagic	non- Hemorrhagic	
Total patients	0.39 (0.36–0.42)	0.52 (0.49–0.54)	<0.001
Gender			
Male	0.37 (0.32–0.43)	0.54 (0.51–0.58)	0.001
Female	0.40 (0.37–0.43)	0.49 (0.46–0.52)	0.008
Age			
Adults (≥18 y)	0.40 (0.37–0.42)	0.52 (0.49–0.54)	<0.001
Children (<18 y)	0.28 (0.16–0.41)	0.51 (0.44–0.57)	0.024
Suzuki Stages			
Suzuki 1–2	0.37 (0.30–0.44)	0.41 (0.38–0.44)	0.258
Suzuki 3–4	0.37 (0.34–0.39)	0.54 (0.51–0.57)	<0.001
Suzuki 5	0.47 (0.37–0.58)	0.63 (0.56–0.71)	0.077

Logistic regression (multivariate analysis) was used to analyze the differences of F-V ratio between hemorrhagic and non-hemorrhagic MMD in different demographic groups.

**Table 3:** Logistic Regression of the Relationship between 10*(F-V Ratio) and Hemorrhagic MMD or MMS.

Factors	P value	OR	95% CI	
			Lower	Upper
Model 1	<0.001	0.344	0.222	0.534
Model 2	<0.001	0.323	0.204	0.510
Model 3	<0.001	0.295	0.182	0.477
Model 4	<0.001	0.275	0.163	0.466
Model 5	<0.001	0.290	0.169	0.500
Model 6	<0.001	0.291	0.164	0.517

Model 1: univariate analysis of 10*(F-V Ratio).

Model 2: adjusted by age and gender on the basis of Model 1.

Model 3: adjusted by Suzuki stage (Suzuki 3–4) on the basis of Model 2.

Model 4: adjusted by the dilation of AchA (grade 2) on the basis of Model 3.

Model 5: adjusted by the dilation of Pcom (grade 2) on the basis of Model 4.

Model 6: adjusted by complicated aneurysm on the basis of Model 5.

**Table 4:** The Different Cut-off Points of F-V Ratio in Predicting Hemorrhagic Stroke in Patients with MMD or MMS.

F-V Ratio	Se %	Sp %	PPV %	NPV %
0.25	2.3	98.6	33.3	76.4
0.35	34.9	92.0	57.7	81.9
0.45	83.7	65.9	43.4	92.9
0.55	95.3	33.3	30.8	95.8
0.65	97.7	15.9	26.6	95.7

F-V Ratio: the ratio of CFCT to CVCT; Se: sensitivity; Sp: specificity; PPV: positive predictive value; NPV: negative predictive value.

**Table 5:** Logistic Regression and ROC curve analysis of two different models for predicting Hemorrhagic MMD or MMS.

	Model 1	Model 2
Logistic Regression		
Nagelkerke R^2^	0.495	0.583
AIC	152.713	125.572
ROC curve analysis		
P value	<0.0001	<0.0001
AUC	0.8748	0.9206
95% CI		
Lower	0.8242	0.8777
Upper	0.9254	0.9636

Model 1: multivariate analysis of demographic and structural metrics (including, gender, Suzuki stage (Suzuki 3–4), dilation of AchA (grade 2), Pcom (grade 2) and complicated aneurysm).

Model 2: multivariate analysis of demographic, structural metrics (including, gender, Suzuki stage (Suzuki 3–4), dilation of AchA (grade 2), Pcom (grade 2) and complicated aneurysm) and the 10*(F-V ratio).

## Discussion

Our study showed a close correlation between the CCT, including the CFCT and CVCT, and subtype of stroke in patients with MMD or MMS. The F-V ratio was inversely correlated with the possibility of hemorrhagic stroke. The model using the F-V ratio and structural metrics was more effective than the model that only used structural metrics for predicting hemorrhagic MMD or MMS.

It is generally recognized that ischemic stroke associated with MMD or MMS occurs with chronic stenosis or occlusion of the ICA or its branches, when compensatory collateral vessels cannot supply adequate blood to the brain^7^. Most cerebral infarction secondary to MMD or MMS is located at the ICA territory (frontal lobe is most common), while PCA involvement is present in 20–30% of MMD, resulting in PCA territory infarction^23, 24^. On the contrary, mainly intracranial hemorrhage occurs due to the rupture of abnormal moyamoya vessels and dilated collateral vessel such as the anterior choroidal artery, posterior communicating artery, and aneurysm^5, 15–19^. In our study, the most common region of infarction was the frontal lobe (88.0%), with 27.9% of patients having PCA involvement. The most common hemorrhagic disorder was intraventricular hemorrhage (49.3%). Hemorrhagic MMD or MMS was more common in adults than children (23:1, P = 0.128), and in females than males (1.4:1, P = 0.191), but without statistical significance. Patients with hemorrhagic MMD are most common in Suzuki stages 3 to 4, and the proportion of abnormal dilation (Grade 2) of AChA and PComA and aneurysm was significantly higher in patients with hemorrhagic MMD than in patients with ischemic MMD. ROC curve analysis of age, gender, Suzuki stage (Suzuki 3–4), dilation of AChA (Grade 2), PComA (Grade 2), and aneurysm for predicting hemorrhagic MMD or MMS showed that the AUC was 0.8748 (P < 0.0001), which is in accordance with the data from some previous studies^15, 17–19, 23, 24^. Interestingly in several patients, we noticed that the dilation of AChA, PComA and aneurysm around the AChA and PChA had regressed or disappeared after extra–intracranial revascularization surgery, which may contribute to the decreased risk of re-bleeding after revascularization (Supplementary Fig. S1).

To the best of our knowledge, a substantial proportion of the population has congenital or acquired dysplasia of the cerebral venous system, with most people exhibiting no symptoms. However, in exceptional circumstances, abnormal cerebral venous drainage can lead to increased intravascular pressure load and abnormal cerebral blood flow, resulting in brain damage. Previous studies have reported that some patients with MMD or MMS have complications of hypercoagulability, which can cause cerebral venous thrombosis and abnormal cerebral venous drainage^25–28^. Cerebral venous drainage in other disorders, such as cerebral hemorrhage, cerebral infarction and arteriovenous malformation, has attracted increasing attention^29–32^. However, the relationship between the cerebral venous system and stroke related to MMD or MMS has been rarely reported.

Previous studies about cerebral venous drainage have mainly been limited to a description of the structure or indirect signs, such as diameter and number of draining veins, or venous reflux^29, 33^. Although structural imaging such as CT venography (CTV), MR venography (MRV), and DSA can reflect the shape of the cerebral venous system, some diseases such as stenosis of internal jugular vein, defects of venous valves, or increased intrathoracic pressure often do not show unusual findings on structural images, but can result in increased intracranial pressure and delayed CCT, especially venous circulation time^34, 35^. Therefore, CCT can more accurately reflect cerebral blood flow, and CVCT is an important indicator of abnormal cerebral venous drainage^36–38^. CCT can help us further understand changes in cerebral hemodynamics in patients with MMD or MMS, which may be of great significance in clinical practice.

Due to the different methods and definitions, the normal CCT of healthy population differs accordingly^35, 39, 40^. In the present study, the mean CTCT (16.54 s) was prolonged in patients with MMD or MMS, compared with healthy population of previous studies^35, 39^. The CFCT of hemorrhagic sides was shorter than non-hemorrhagic sides, with a significant statistical difference (P < 0.001). Presumably, since hemorrhagic MMD was often in Suzuki 3 or Suzuki 4 stage, moyamoya vessels were relatively rich, and collateral arteries were dilated significantly, cerebral blood filling is faster in hemorrhagic MMD than non-hemorrhagic MMD, which coincided with above-mentioned results of this study and previous studies^15, 17, 18^. However, the CVCT was longer in the hemorrhagic sides than non-hemorrhagic sides, also with a significant difference (P < 0.001), suggesting poor cerebral venous drainage is probably a risk factor of hemorrhagic stroke in the patients with MMD or MMS. Subgroup analyses of different Suzuki stages, however, showed that this phenomenon was primarily apparent in the hemispheres between Suzuki 3 and Suzuki 4, with the exception of early stages (Suzuki 1 to Suzuki 2) or late stages (Suzuki 5). It is well understood that cerebral vessels of MMD are approximate to normal condition during Suzuki 1 and Suzuki 2 stages, and internal carotid arteries are approximate to occlusion during Suzuki 5 and Suzuki 6 stages, with relatively low intravascular pressure load, so it is of less possibility of bleeding during these stages.

Many other factors can affect CCT such as blood pressure, heart rate, and other parameters of angiography. In addition, CFCT and CVCT may correlate with each other. To diminish the influential factors of CCT, the F-V ratio was used to analyze the differences in CCT between hemorrhagic and non-hemorrhagic MMD or MMS. The results indicated that the F-V ratio was inversely correlated with the possibility of hemorrhagic stroke. The ROC curve analysis of the F-V ratio showed that the best cut-off point for differentiating hemorrhagic MMD or MMS from non-hemorrhagic MMD or MMS was 0.4344 (sensitivity = 81.4%, specificity = 73.2%, AUC = 0.8016), according to Youden’s index (Figs 2 and 3). The smaller the F-V ratio, the lower the sensitivity and higher the specificity for predicting hemorrhagic stroke (Table 4). The model using structural metrics and the F-V ratio was superior to the model that only used structural metrics for predicting hemorrhagic MMD or MMS (P = 0.022) (Table 5, Fig. 2).

From our perspective, this phenomenon can be attributed to two mechanisms: the rapid filling of cerebral circulation and dysfunction of cerebral venous drainage. The rapid filling is probably relevant to substantial moyamoya vessels, significantly dilated and extended collateral arteries, such as anterior choroidal artery and posterior communicating artery. For the latter mechanism, there are many factors or diseases that can lead to dysfunction of cerebral venous drainage, including intracranial and extracranial factors. Intracranial factors, such as cerebral venous sinus thrombosis and stenosis of cerebral venous sinus, can directly cause impaired outflow of intracranial blood circulation, leading to disorders of cerebral venous drainage and increased intravascular pressure load. Extracranial factors, including stenosis of internal jugular veins, defects of venous valves, or increased intrathoracic pressure, can also result in incremental intravascular pressure load by inducing increased pressure of outlet of cerebral circulation. Eventually, the incremental intravascular pressure load can increase the opportunity of rupture of fragile moyamoya vessels, dilated collateral arteries or aneurysms, which is similar with other cerebral vascular malformation^29, 30, 33^.

The limitations of our study should be mentioned. First, all of the patients in this study were enrolled from a single center, so potential selection bias is inevitable. However, we tried to reduce in-house selection bias as much as possible by collecting patients consecutively. Second, the nature of case-control studies necessitates further prospective cohorts or randomized studies to confirm our conclusions.

## Conclusions

The rapid filling and poor venous drainage of cerebral blood circulation are probably risk factors of hemorrhagic stroke secondary to MMD or MMS. The F-V ratio, in addition to age, gender, Suzuki stage, dilation of AChA and PComA and aneurysm, may be used in clinical practice to identify high-risk individuals of hemorrhagic stroke, who need preventive revascularization surgery before the occurrence of bleeding and avoidance of antiplatelet agents.

## Electronic supplementary material

Supplementary Information
