# Supplementary material for: Difference in Cerebral Circulation Time between Subtypes of Moyamoya Disease and Moyamoya Syndrome
Source: Sci Rep. 2017 May 31;7:2587. doi: 10.1038/s41598-017-02588-1 (PMC5451479; doi:10.1038/s41598-017-02588-1)
Supplement: Supplementary file 1 — Supplementary Information [file 41598_2017_2588_MOESM1_ESM.pdf]

Supplementary Information

Difference in Cerebral Circulation Time between Subtypes of Moyamoya Disease and Moyamoya Syndrome

Kaijiang Kang, MD [1*]; Jingjing Lu, MD [1*]; Dong Zhang, MD[2]; Youxiang Li, MD[3]; Dandan Wang, MD[1]; Peng Liu, MD[3]; Bohong Li, MD[1]; Yi Ju, MD[1]; Xingquan Zhao, MD[1]

[1]Department of Neurology, Beijing Tiantan Hospital, Capital Medical University, China National Clinical Research Center for Neurological Diseases, Center of Stroke, Beijing Institute for Brain Disorders, Beijing, China.

[2]Department of Neurosurgery, Beijing Tiantan Hospital, Capital Medical University, Beijing, China.

[3]Department of Neurosurgery, Beijing Neurosurgical Institute, Capital Medical University, Beijing, China.

* These authors contributed equally to the manuscript.

Corresponding authors: Prof. Xingquan Zhao, Department of Neurology, Beijing Tiantan Hospital, Capital Medical University, No.6 Tiantanxili, Dongcheng District, Beijing, China, 100050 (tel.: 86-10-67098471; fax: 86-10-67013383; E-mail: zxq@vip.163.com); Prof. Yi Ju, Department of Neurology, Beijing Tiantan Hospital, Capital Medical University, No.6 Tiantanxili, Dongcheng District, Beijing, China, 100050 (tel:86-10-67098330, fax: 86-10-67013383, E-mail: juyi1226@163.com).

Number of supplementary tables: 3

Number of supplementary figures: 1

Supplementary Table S1

Basic demographic information and neuroradiological characterizations: Gender, Age, Distribution of Suzuki stages, Grades of AChA, PComA, PCA, ECA, and associated aneurysms in each hemispheric group.

	Subtypes of MMD or MMS		Total Number	P value
	Hemorrhagic	non- Hemorrhagic		
Gender				0.191
Male	30 (25.2%）	89 (74.8%)	119 (47.8%)	
Female	41 (31.5%）	89 (68.5%)	130 (52.2%)	
Age				0.128
Children (＜18 y)	3 (10.7%)	25(89.3%)	28(11.2%)	
Adults (≥18 y)	68 (30.8%)	153 (69.2%)	221(88.8%)	
Suzuki Stage				0.179
Suzuki 1	2 (13.3%)	13 (86.7%)	12 (6.0%)	
Suzuki 2	5 (13.2%)	33 (86.8%)	38 (15.3%)	
Suzuki 3	21 (47.7%)	23 (52.3%)	44 (17.7%)	
Suzuki 4	31 (34.8%)	58 (65.2%)	89 (35.7%)	
Suzuki 5	10 (22.2%)	35 (77.8%)	45 (18.1%)	
Suzuki 6	2 (11.1%)	16 (88.9%)	18 (7.2%)	
AchA Grading				< 0.001
0	9（11.8%）	67（88.2%）	66（30.5%）	
1	12（20.7%）	46（79.3%）	58（23.3%）	
2	48（60.8%）	31（39.2%）	79（31.7%）	
3	2（5.6%）	34（94.4%）	36（14.5%）	
PcomA Grading				0.001
0	22（25.0%）	66（75.0%）	88（35.3%）	
1	2（5.6%）	34（94.4%）	36（14.5%）	
2	41（51.9%）	38（48.1%）	79（31.7%）	
3	6（13.0%）	40（87.0%）	46（18.5%）	
PCA Grading				0.850
0	14（27.5%）	37（72.5%）	51（20.5%）	
1	57（28.8%）	141（71.2%）	198（79.5%）	
ECA Grading				0.887
0	32（28.1%）	82（71.9%）	114（45.8%）	
1	39（28.9%）	96（71.1%）	135（54.2%）	
Aneurysms				< 0.001
Yes	19 (82.6%)	4 (17.4%)	23 (9.2%)	
No	52 (23.0%)	174 (77.0%)	226 (90.8%)	
Total Number	71（28.5%）	178（71.5%）	249（100%）	

Supplementary Table S2

Clinical features: sites of hemorrhage and infarction of the patients in the study.

	Frequency (n)	Percentage (%)
Sites of Bleeding		
IVH	35	49.3%
ICH+IVH	14	19.7%
ICH	14	19.7%
SAH	8	11.3%
Sites of Infarction		
Frontal lobe	139	88.0%
Parietal lobe	22	13.9%
Temporal lobe	20	12.7%
Occipital lobe	16	10.1%
Basal ganglia	30	19.0%

Supplementary Table S3

The Difference of F-V Ratio between Hemorrhagic Sides and non-Hemorrhagic Sides in the Same Patient.

Patient	F-V Ratio	
	Hemorrhagic	non-Hemorrhagic
1	0.60	0.40
2	0.42	0.50
3	0.43	0.44
4	0.41	0.56
5	0.33	0.38
6	0.42	0.50
7	0.28	0.33
8	0.43	0.55
9	0.36	0.35
10	0.54	0.59
11	0.40	0.44
12	0.42	0.44
13	0.33	0.52
14	0.36	0.47
15	0.47	0.44
16	0.29	0.41
17	0.31	0.48
18	0.23	0.38
19	0.32	0.47

Paired-sample nonparametric test (Wilcoxon Signed Ranks Test) was used; $P=0.005$.

Supplementary Figure S1

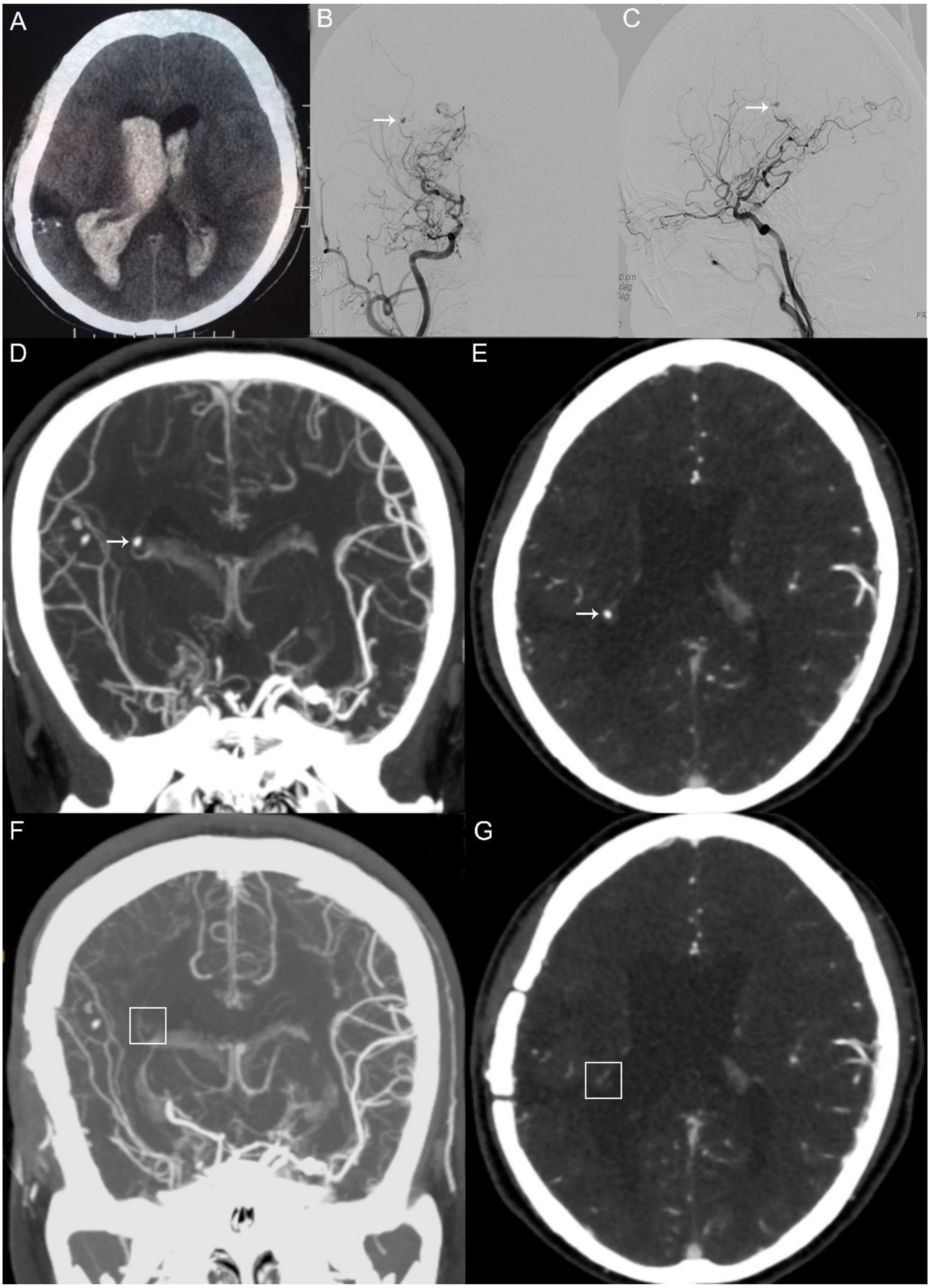

A 39-year-old female patient with hemorrhagic moyamoya disease. CT showed bilateral (mainly in the right side) intraventricular hemorrhage (A). DSA exhibited dilation of right AChA, complicated with an aneurysm at the distal segment (B, C). CTA also revealed the aneurysm

of anterior choroidal artery on the right side (D, E). The patient was followed up for 3 months after direct revascularization without rebleeding, and the CTA (3 months after the surgery) suggested that the aneurysm around the right anterior choroidal artery had almost disappeared(F,G).
